# Preferential Binding of Polyphenols in Blackcurrant Extracts with Milk Proteins and the Effects on the Bioaccessibility and Antioxidant Activity of Polyphenols

**DOI:** 10.3390/foods13040515

**Published:** 2024-02-07

**Authors:** Ting Mao, FNU Akshit, Iresha Matiwalage, Subha Sasidharan, Caren Meyn Alvarez, Philip Wescombe, Maneesha S. Mohan

**Affiliations:** 1 Alfred Dairy Science Laboratory, Department of Dairy and Food Science, South Dakota State University, Brookings, SD 57007, USA; ting.mao@sdstate.edu (T.M.); akshit.lnu@sdstate.edu (F.A.); 2 Department of Wine, Food and Molecular Biosciences, Lincoln University, Lincoln 85084, New Zealand; iresha.matiwalage@lincolnuni.ac.nz (I.M.); subha.sasidharannair@lincolnuni.ac.nz (S.S.); carenmeyn@gmail.com (C.M.A.); philip.wescombe@oceaniadairy.co.nz (P.W.); 3 Yili Innovation Center Oceania, Lincoln University, Lincoln 85084, New Zealand; 4 National Center of Technology Innovation for Dairy, Hohhot 010000, China

**Keywords:** anthocyanins, caseins, whey proteins, bioavailability, antioxidant

## Abstract

Milk proteins are well-known delivery agents; however, there is no clear understanding of the competitive interactions of milk proteins with polyphenols in mixed complex systems. Here, we investigate the preferential competitive interactions of different polyphenols present in blackcurrant extract with milk proteins by quantifying the protein-bound polyphenols and comparing the factors affecting these interactions. In addition, bioaccessibility and antioxidant activity were studied after *in vitro* gastric digestion. Our results indicated that polyphenols from blackcurrant extracts were preferentially bound to caseins more than whey proteins, with noncovalent interactions causing secondary structural changes in the protein. The hydrophobicity and the charge of the polyphenols were negatively and positively related to the number of polyphenols bound to casein and whey proteins, respectively. Moreover, the bioaccessibility and antioxidant activity of polyphenols were enhanced in the presence of milk proteins in milk-based blackcurrant samples when compared to polyphenol and protein-alone samples in the *in vitro* gastric phase. These findings underscore the critical role of milk proteins in encapsulating or delivering polyphenols. This will pave the way for boosting the bioavailability of polyphenols by complexing them with milk proteins and formulating functional dairy foods, integrating the beneficial effects of these compounds.

## 1. Introduction

Polyphenols are naturally present in fruits, vegetables, cereals, seeds, and herbs as secondary metabolites. They play major roles in defending plants against internal or external stresses such as free radicals, ultraviolet radiation, fungi, insects, and animals [1,2,3]. The polyphenol classifications include flavonoids, lignans, stilbenes, phenolic acids, and other subclasses, such as tannins and lignins, and they give plants their scent, color, and flavor. Investigations about the health benefits of polyphenols for humans have led to discoveries about their diverse biological effects, including antioxidant, anti-inflammatory, anti-viral, anti-proliferative, and hormone-regulatory properties [4]. The long-term consumption of polyphenols has been shown to mitigate the effects of aging, infections, asthma, and the onset of a variety of chronic diseases, including cardiovascular diseases, hypertension, type 2 diabetes, and cancer [2,5]. Hence, polyphenol-rich foods are recommended as an essential component of the diet [6]. However, the claims about the health benefits of polyphenols for food and pharmaceutical industry products have been limited owing to the rapid degradation rate and low bioavailability of polyphenols from diet in the human digestive system. For example, several researchers reported a marked 50–85% reduction in the bioavailability of polyphenols (especially anthocyanins) during the digestion process, compared with non-digested samples [7,8,9,10].

Polyphenols can interact with milk proteins to form complexes via noncovalent interactions and conjugates via covalent interactions, leading to changes in the functional, nutritional, and biological activities of polyphenols as well as milk proteins [11]. Blackcurrant is abundant in polyphenols, especially anthocyanins [12], and their interaction with whey proteins has been reported to increase the bioaccessibility of polyphenols [13,14,15]. However, there are no studies about the competitive binding of different polyphenols from blackcurrant extracts with different types of milk proteins (caseins, whey proteins, and the combination of all milk proteins) and the nature and sites of these binding interactions. The effects of these interactions on the bioaccessibility and antioxidant activity of polyphenols from blackcurrant extract are also not understood. Therefore, our study investigated the free and protein-bound blackcurrant polyphenols after their interaction with milk proteins in aqueous buffered solutions. Subsequently, the degree to which polyphenol properties (molecular weight, logP, hydroxyl groups, pKa, and charge) impacted the differential polyphenol binding to different milk protein fractions was determined using correlation analysis. We also assessed the binding forces in milk protein–blackcurrant polyphenol complexes and explored the effects on bioaccessibility and antioxidant activities of polyphenols using an *in vitro* gastric digestion model. 

## 2. Materials and Methods

### 2.1. Materials

Commercially available pasteurized skim milk (Pams NZ, 3.2% protein content), sodium caseinate 180 (protein content of 92%, Fonterra, Auckland, New Zealand), whey protein isolate (WPI; protein content of 97%; Fonterra, Auckland, New Zealand), and water-extracted blackcurrant extract (XBC 19-09, 319 mg total phenolic content per gram extract, Oxi-fend, Blenheim, New Zealand) were used in this present study. Polyphenol standards, including rutin, cyanidin-3-rutinoside, gallic acid, delphinidin-3-rutinoside, cyanidin-3-sophoroside, protocatechuic acid, quercetin, catechin, epicatechin, epicatechin gallate, epigallocatechin gallate, cyanidin-3-glucoside, kaempferol, ferulic acid, p-coumaric acid, caffeic acid, and procyanidin B1, and solvents used for HPLC were HPLC grade purchased from Sigma-Aldrich New Zealand Co. (Auckland, New Zealand). All the other standards, chemicals, and solvents were analytical grade.

### 2.2. Sample Preparation

#### 2.2.1. Polyphenol Stock Solutions

The stock solution for commercial blackcurrant extract was prepared at a concentration of 50 mg/mL of polyphenols (based on Oxi-fend specification sheet) in phosphate buffer (sodium chloride 1.6% *w*/*v*, potassium chloride 0.04% *w*/*v*, disodium phosphate 0.288% *w*/*v*, monopotassium phosphate 0.048% *w*/*v*) and then was adjusted to pH 6.5 (using 1 M hydrochloric acid), to imitate the milk system. The process involved stirring the powder in PBS solution overnight using a magnetic stirrer at low temperatures using an ice slush bath. It was then centrifuged at 4696× *g* for 20 min at 20 °C. The supernatant was collected by pouring the top layer into another container, and the pH was adjusted to 6.5 using 0.1 N sodium hydroxide and stored at 4 °C.

#### 2.2.2. Casein and Whey Protein Solutions

The stock solutions of caseins and whey proteins were prepared with the composition of casein and whey proteins as present in milk for the individual protein stock solutions (approximately 80% of the total milk protein is caseins, and the rest of the 20% is whey proteins). The casein stock solutions contained 2.81% sodium caseinate 180 dissolved in PBS buffer; the pH was adjusted to 7.5 using 0.1 N sodium hydroxide and stirred overnight, maintaining low temperatures using an ice slush for complete dissolution. After completely dispersing the proteins, the pH of the sodium caseinate solution was decreased to 6.5 using 0.1 N hydrochloric acid. The whey protein stock solution was prepared with 0.7% of WPI dissolved in PBS buffer adjusted to pH 6.5.

#### 2.2.3. Milk Protein–Polyphenol Systems

Two polyphenol-to-protein ratios (5:40 and 10:40) were evaluated in the study. These ratios were identified after comparison with a study conducted by Felix da Silva et al. [16] with a ratio of 1:40 (24 mg of phenolic content per 1 g of protein), yielding very low phenolic content after extraction, which could be lower than the threshold of detection for HPLC analysis for many of the polyphenols. The milk-based blackcurrant samples contained 7.4% and 14.8% (*v*/*v*) polyphenol stock solution mixed in pasteurized skim milk (3.2% protein content) to obtain the targeted 5:40 and 10:40 polyphenol-to-protein ratios, respectively. The casein-based blackcurrant samples comprised 6.1% and 12.2% (*v*/*v*) polyphenol stock solution in caseinate stock solution to achieve polyphenol-to-casein ratios of 5:40 and 10:40, respectively. For the whey protein-based blackcurrant samples, 1.6% and 3.2% (*v*/*v*) of polyphenol stock solutions were made with whey protein stock solution to obtain polyphenol-to-protein ratios of 5:40 and 10:40, respectively. To achieve maximum interaction of the polyphenols with the milk proteins, these solution systems were mixed using a magnetic stirrer for 5 min and then placed in a water bath shaker, maintained at 28 °C for 1 h [17]. The control samples without proteins contained 7.4% and 14.8% (*w*/*v*) of polyphenol stock in PBS buffer at pH 6.5 to mimic the concentration of polyphenols in the milk-based solutions. The samples were then frozen, freeze-dried, and stored for analysis. The samples studied in this experiment include polyphenol and protein-alone control samples and protein (casein, whey protein, and milk)-based blackcurrant samples with polyphenol-to-protein ratios of 5:40 and 10:40.

### 2.3. Loading Efficiency Studies

Loading efficiency studies were conducted to quantify the amount of polyphenols bound to the individual milk protein fractions, casein and whey protein, and the amount of unbound polyphenols. The freeze-dried samples with a concentration of 33.3% (*w*/*v*, g/mL) were prepared by stirring for 1 h on a magnetic stirrer using RO water (5 g powder mixed with 1.5 mL of RO water). The pH was reduced to 4.6 using 0.1 N hydrochloric acid to facilitate casein precipitation, and the supernatant was separated via centrifugation at 4696× *g* for 30 min at room temperature (Supernatant 1 or S_1_). A part of S_1_ was then mixed with 10% trichloroacetic acid (TCA) by vortexing for a minute to separate the remaining suspended whey proteins via centrifugation at 4696× *g* for 30 min at room temperature and collected the supernatant (Supernatant 2 or S_2_). Furthermore, S_1_ and S_2_ were subjected to the acetone extraction procedure for HPLC analysis. Briefly, the acetone-based extraction involved mixing 0.5 mL of supernatant with 1.7 mL 100% acetone by vortexing for 2 min and then stirring for one hour at 40 °C [16] on a shaking water bath with intermittent vortexing. The acetone-extracted polyphenols were separated via centrifugation at 4696× *g* at 4 °C for 30 min. The supernatant collected was vortexed for 1 min with 10% TCA and centrifuged again at 4696× *g* at 4 °C for 30 min to precipitate and remove any solvent-extracted proteins or peptides. This supernatant was removed using a Pasteur pipette, filtered (0.4 micron), and stored at −20 °C for HPLC analysis, total phenolic content, and antioxidant activity.

The quantification of the loading efficiency of polyphenols to the different milk protein fractions (caseins and whey proteins) in milk, casein, and whey protein-based blackcurrant samples has been further explained with the equations below.

For milk-based blackcurrant samples,

Polyphenols bound to casein and whey proteins = A − S_2_, (1)

Polyphenols bound to casein = A − (S_1_ + S_2_), (2)

For casein-based blackcurrant samples,

Polyphenols bound to casein = B − S_2_, (3)

For whey protein-based blackcurrant samples,

Polyphenols bound to whey protein = C − S_2_, (4)

where A, B, and C represent the total polyphenol content in the milk-, casein-, and whey protein-based blackcurrant samples, respectively. S_1_ represents polyphenols bound to whey protein, and S_2_ represents free polyphenols in milk-, casein-, or whey protein-based blackcurrant samples.

### 2.4. Solvent-Based Extraction Procedure for HPLC

The acetone-based extraction procedure has been modified from the method by Cebeci and Şahin-Yeşilçubuk [18]. Briefly, 2 g of the freeze-dried samples were weighed and mixed with 20 mL of 70% acetone using a vortex mixer for 5 min. These acetone-added samples were then placed in a water bath shaker for 1 h at 40 °C with intermittent vortexing. The temperature of mixing was chosen to decrease the affinity of the polyphenols to milk proteins [17] and ensure better extractability. The extracts were obtained via centrifugation at 4696× *g* for 30 min at 4 °C and collecting the supernatant using a Pasteur pipette. The supernatant extracted was mixed with 8% TCA and vortexed for 1 min [19]. The extracts were then centrifuged at 4696× *g* for 30 min at 4 °C. The supernatant was collected using a Pasteur pipette and stored frozen for analysis.

### 2.5. HPLC Quantification of Polyphenols

The filtered (0.4 micron) acetone extracts of samples were analyzed using an Agilent 1200 series Gradient HPLC, comprising a quaternary pump, DAD, and FLD detectors. The column used was ACE 3μ C18-PFP 150 × 4.6 mm (EXL-1110-1546U, Advanced Chromatography Technologies, Aberdeen, Scotland) with the column maintained at 20 °C. The solvent systems were A (0.05 M NH_4_H_2_PO_4_, pH 2.6), B (100% Acetonitrile), and C (0.2 M H_3_PO_4_, pH 1.5). The elution system was as follows: 0–2 min 100% A, 2–5 min 93.6% A and 6.4% B, 5–17 min 2.8% A and 11.2% B, 17–22 min 3.6% A and 14.4% B, 22–29.5 min 4.2% A and 16.8% B, 29.5–55 min 6.6% A and 26.4% B, 55–70 min 10% A and 40% B, 70–75 min 10% A and 40% B, 75–78 min 36% A and 64% B, 78–81 min 36% A and 64% B, 81–90 min 100% A. The flow rate was maintained at 0.8 mL/min with an injection volume of 10 μL. Detection was carried out at 280 nm, 320 nm, 360 nm, and 520 nm. FLD was used to identify procyanidin B1 and B2, catechin, and epicatechin. Seventeen standards were scanned from 220 to 600 nm, and the spectrum was stored for evaluation and comparison of compounds. A concentration of 1000 ppm was utilized for the preparation of each individual standard. These standards were then combined in equal proportions to create a mixed standard with a concentration of 58.8 ppm for each constituent. The calibration curve for each standard was established at concentrations of 100 ppm, 58.8 ppm, 29.4 ppm, 14.71 ppm, 7.35 ppm, and 3.675 ppm. All calibration curves had an R^2^ greater than 0.99. 

### 2.6. Fourier-Transform Infrared (FT-IR) Spectra

The Fourier-transform infrared (FT-IR) spectra were obtained using a Nicolet 6700 FT-IR spectrometer (Thermo Fisher Scientific Inc., Waltham, MA, USA) at room temperature. Each spectrum was recorded at the absorbance between 400 to 4000 cm^−1^ at a resolution of 4 cm^−1^ using an average of 60 scans by Nicolet Omnic 32 software (Thermo Fisher Scientific Inc., Waltham, MA, USA). The measurements were made directly for freeze-dried powder samples for milk proteins, whey protein, sodium caseinate 180, polyphenol extract, and casein-, whey protein-, and milk-based blackcurrant samples. Tests were carried out in triplicate with three measurements for each replicate. To determine the effects of polyphenols on the secondary structure of milk proteins, the spectral region between the wavenumbers of 1600 cm^−1^ and 1700 cm^−1^ was selected. The full bandwidth at half height (FWHH) was adjusted to 13 cm^−1^, and the resolution enhancement factor was set to 2.4. The selected spectra were smoothed with 2 points, and the second derivative calculations were used to obtain the percentage of secondary structural elements by the PeakFit Version 4.12 software (SPSS Inc., Chicago, IL, USA).

### 2.7. In Vitro Bioaccessibility in the Gastric Phase

The bioaccessibility of freeze-dried milk-based blackcurrant samples in the gastric phase was assessed under conditions with modification to the method by Kaur et al. [20]. Samples (1.5 g; equivalent to 70 mg of Nitrogen) were stirred using a magnetic stirrer in 17 mL of 0.1 M HCl (pH 1.9 ± 0.1) in closed containers for the time till temperature acquired to 37 °C (~5 min). Aliquots of 1 mL were collected from this mixture for further analysis and designated as ‘5 min’ time point samples. Pepsin solution (2.5 mL containing 0.7 mg of pepsin in 0.1 M HCl with pepsin enzyme activity ~2500 Units/mg) was then added to the solution. After 1 h of incubation with pepsin (pH 1.9 ± 0.1; simulated gastric digestion), aliquots of 1 mL were taken from the digests (with the enzymes) after 30 and 60 min of gastric digestion. The pH of the aliquots was adjusted to 8 using 0.1 N sodium hydroxide by using pH papers, to arrest the activity of the pepsin. Centrifuged the collected aliquots from different digestion time points at 4000× *g* for 15 min, and the clear supernatants were frozen and stored for further analysis. All samples were digested in triplicates. 

### 2.8. Total Phenolic Content Measurement

Measurement of the total phenolic content (TPC) was carried out similarly to the methods described by Huang et al. [21] and Archaina et al. [22] with minor modifications using Folin Ciocalteu’s phenol reagent. Gallic acid was used as standard, and the TPC of the sample was expressed as mg per g of gallic acid equivalent (GAE). Frozen samples from acetone extraction and *in vitro* digestion (not acetone-extracted) were defrosted at room temperature and centrifuged (Hettich Zentrifugen Rotina 380 centrifuge, Tuttlingen, Germany) at 4000× *g* for 15 min. Supernatants were collected from samples and then diluted with RO water to fall within the standard curve. Gallic acid standard solutions between 0 and 250 μg/mL in 70% methanol or 70% acetone were prepared for the standard curve, depending on whether the samples were *in vitro* digests or acetone extractions, respectively. About 20 μL of standard solutions or diluted samples were loaded in a 96-well microwell plate, followed by the addition of 100 μL of 0.2 N Folin Ciocalteu reagent and 80 μL of 7.5% Sodium carbonate, which were all thoroughly mixed in using the pipette. The blank comprised 20 μL 70% methanol or 70% acetone, depending on the type of sample, and 200 μL PBS (pH 7.4) buffer. Acetone-based extracts and *in vitro* digest samples and standards in the microplate were covered with aluminum foil and incubated for 2 h at room temperature in a dark place before measuring the absorbance by using a spectrophotometer at 760 nm. All tests were performed in triplicate in polypropylene microplates for acetone solvent compatibility with the material.

### 2.9. The ABTS (2,2′-Amino-Di (-Ethyl-Benzothiazoline Sulphonic Acid-6) Ammonium Salt) Antioxidant Assay

The ABTS radical-scavenging test was conducted using the method of Wang et al. [23] with some modifications. For the ABTS stock solution, 9.5 mL of 7 mM ABTS was mixed with 245 μL of 100 mM potassium persulphate (K_2_S_2_O_8_) stock solution and made up to 10 mL with RO water, covered with foil to protect from light and kept overnight for reaction in the dark at room temperature. The next day, the ABTS radical solution was diluted with phosphate-buffered saline (PBS, pH 7.4) up to an absorbance of 0.70 (+0.02) at 734 nm. Sample (20 μL, diluted *in vitro* digests or acetone-based extracts) and 200 μL of diluted ABTS radical solution were mixed in the microplates and incubated for 6 min at room temperature. The blank samples contained 20 μL 70% methanol or 70% acetone, depending on the type of sample (*in vitro* digests or acetone-based extracts, respectively), and 200 μL PBS (pH 7.4) buffer. The absorbance of the reaction mixture was measured at 734 nm. The common practice of using the Trolox standard curve to calculate the antioxidant activity was not used in this study as the Trolox-alone standards do not accommodate the effect or interference of milk proteins and interactions of polyphenols with milk proteins on the antioxidant assay. Hence, the degree of antioxidant activity was represented as inversely proportional to the absorbance measured. 

### 2.10. Statistical Analyses

The statistical analysis was carried out using IBM SPSS statistics. Results are reported as mean ± standard deviation (SD). ANOVA was performed using the General Linear Model, and mean separation was identified using Tukey’s test, with differences between treatments and samples considered statistically significant with a *p* value of less than 0.05. Pearson’s correlation analysis was performed using OriginPro 2024 (OriginLab Corporation, Northampton, MA, USA) to evaluate the relationship of the properties of polyphenols (logP, molecular weight, and pKa) with the quantity of polyphenols bound to caseins, whey proteins, and milk proteins, from the loading efficiency studies.

## 3. Results and Discussion

### 3.1. Total Phenolic Content and Polyphenol Profile of Blackcurrant Extract and Milk Protein-Based Samples

**Figure 1:**
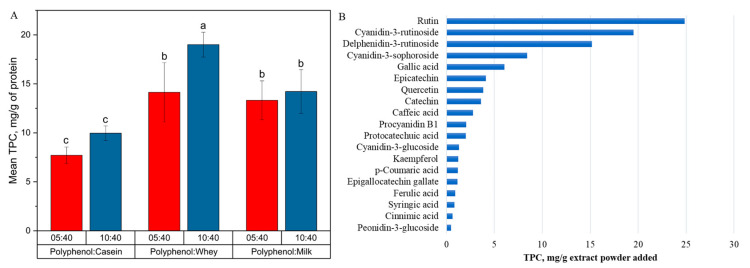
(**A**): the total phenolic content (TPC) of casein-, whey protein-, and milk-based blackcurrant samples with polyphenol-to-protein ratios of 5:40 and 10:40, expressed as mg gallic acid equivalent (GAE) per gram protein added. Different letters above the error bars (standard deviation) represent a significant difference between groups. (**B**): polyphenol profile of the freeze-dried Oxi-fend commercial blackcurrant aqueous extract.

The TPC analysis conducted provides an estimate of the total polyphenolic content in the milk protein-based blackcurrant samples and polyphenol-alone control samples. The TPC was 217.02 ± 12.75 mg of GAE per g of freeze-dried blackcurrant powder (control), which was lower than the manufacturer specification (319 mg total phenolic content per gram extract) for the commercial extract. This is owing to the further buffer-based extraction conducted in this study and differences in the solvent-based extraction procedure utilized in our study compared to the manufacturer. The major polyphenols in one gram of freeze-dried blackcurrant powder were rutin (24.85 mg), cyanidin-3-rutinoside (19.53 mg), delphinidin-3-rutinoside (15.17 mg), cyanindin-3-sophoroside (8.41 mg), gallic acid (6.05 mg), and epicatechin (4.11 mg) (Figure 1). The most abundant polyphenols in the blackcurrant extracts were anthocyanins, including cyanidin-3-rutinoside, delphinidin-3-rutinoside, cyanindin-3-sophoroside, and cyanindin-3-glucoside. However, the amount was less than the data reported by Jurgoński et al. [24], who extracted polyphenols from blackcurrant pomace using a solution composed of methanol, water, and formic acid with a volume ratio of 50:48:2. In our analysis, 70% acetone was used to extract polyphenols from aqueous phase extracted Oxi-fend commercial blackcurrant extracts. Given that these extracts are water-based, certain polyphenols could have been lost in the production process of these extracts. 

A notable significant difference was observed in the amount of polyphenols extracted from whey protein-based blackcurrant samples at different polyphenol-to-protein ratios. On increasing the polyphenol concentration in samples from polyphenol-to-protein ratios of 5:40 to 10:40, the total extractable polyphenol content increased from 14.14 ± 3.02 to 19.00 ± 1.27 mg of GAE per g of protein added (*p* < 0.0001). No significant difference was observed in the amount of polyphenols extracted from casein-based blackcurrant samples with different polyphenol-to-protein concentration ratios, although a higher ratio contributed to slightly higher total polyphenol extracted (8.84 ± 1.39 mg of GAE per g protein added; *p* = 0.129). Similarly, there was no difference in the TPC extracted from milk-based blackcurrant samples containing both the casein and whey protein fractions in combination, with different polyphenol-to-milk ratios (13.77 ± 2.10 mg of GAE per g protein added; *p* = 0.908). The similarity between the results of casein- and milk-based blackcurrant samples is likely due to the fact that the majority of the protein fraction in milk comprises caseins, at around 80% of the total protein content.

The extraction of polyphenols (as measured by TPC) from protein-based blackcurrant samples was highest from whey-, followed by milk-, and then casein-based blackcurrant samples when compared at the same polyphenol-to-protein concentration ratio. This observation is attributed to the stronger interaction of polyphenols with caseins, which form unique large porous structures known as casein micelles [25], which affects the extractability of the polyphenols from the caseins compared to the easier extraction of polyphenols from the whey proteins in the whey-based blackcurrant samples. The high proline content in caseins also contributes to their higher affinity to polyphenols than whey proteins, as more hydrophobic sites were available for interaction with polyphenols [26]. For instance, β-casein, which contained the highest proline content in milk proteins (16.7% of 209 amino acid residues) [27], exhibited the highest binding affinity to (−)-epigallocatechin gallate (EGCG), (−)-epigallocatechin (EGC), and (−)-epicatechin (EC) in the gastric environment (pH 2.0) compared to β-lactoglobulin [28]. However, the type of proteins that interact with polyphenols is not the only factor affecting the binding affinity. The solution environment, including pH, temperature, and polyphenol characteristics, also needs consideration. For example, chlorogenic acid showed higher affinity to β-lactoglobulin compared to α-, β-, and κ-casein at 25 °C and 37 °C [29]. 

In summary, the main polyphenols in the blackcurrant extract were found to be anthocyanins, and the total extractable polyphenols from whey protein-based blackcurrant samples were the highest, followed by milk- and casein-based blackcurrant samples. These results demonstrate the possibility of modifying the amount of extractable polyphenols via binding with milk proteins with potential application in bioavailability control.

### 3.2. Preferential Binding and the Competition of Polyphenols for Binding with Milk Proteins

**Figure 2:**
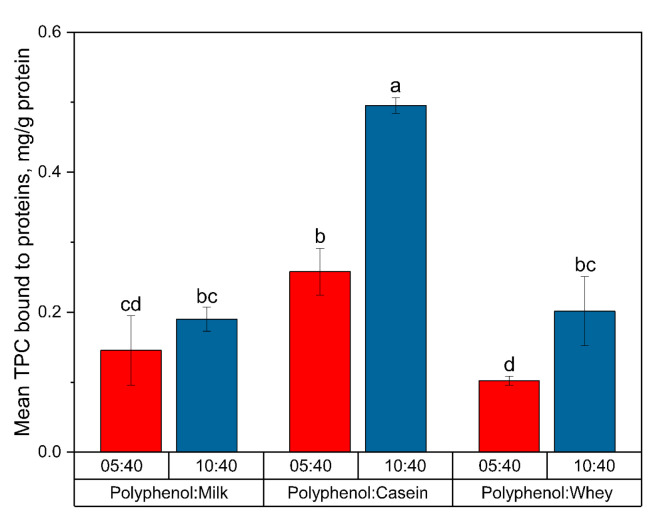
The total phenolic content (TPC) of polyphenol bound to casein-, whey protein-, and milk-based blackcurrant samples with polyphenol-to-protein ratios of 5:40 and 10:40. Different letters above the error bars (standard deviation) represent a significant difference between groups.

The loading efficiency of the polyphenols to the milk proteins in the different samples was compared as TPC (expressed as mg of GAE per g protein) to each milk protein fraction in the sample (Figure 2). The whey protein-based blackcurrant samples exhibited a notable increase in the amount of polyphenols bound to the proteins for the higher polyphenol-to-protein ratios of 10:40 (0.20 ± 0.05) compared to 5:40 (0.10 ± 0.01 mg of GAE per g protein added, respectively; *p* = 0.028). For the casein-based blackcurrant samples, there was a difference in the content of polyphenols bound to the caseins when mixed at different concentrations, with 0.26 ± 0.03 and 0.50 ± 0.01 mg of GAE per g protein added for samples with polyphenol-to-protein ratios of 5:40 and 10:40, respectively (*p* < 0.0001). However, no significant results were observed when increasing the polyphenol-to-protein ratio from 5:40 to 10:40 for the milk-based blackcurrant samples (0.16 ± 0.04 mg of GAE per g protein added; *p* = 0.575). By looking into the specific protein fraction bound to milk proteins, we found that around 7.8% of the polyphenols bound to whey proteins (0.01 ± 0.002 mg of GAE per g protein added) at the low polyphenol-to-protein ratio of 5:40. After increasing the polyphenol concentration, the proportion of polyphenols bound to whey proteins increased to 26.1% (0.05 ± 0.008 mg of GAE per g protein added). This is opposed to casein-bound polyphenols, which increased relatively less from 0.13 ± 0.04 to 0.14 ± 0.01 mg of GAE per g protein added for the polyphenol-to-protein ratio of 5:40 to 10:40, respectively. Hence, the competitive binding of polyphenols to different fractions of milk proteins (e.g., caseins vs. whey) may lead to binding saturation occurring at a lower polyphenol-to-protein ratio (5:40).

**Figure 3:**
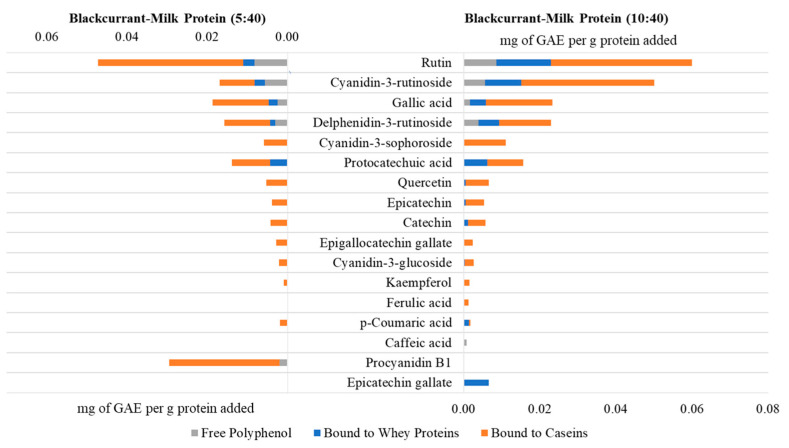
Free and bound polyphenol content (expressed as mg of gallic acid equivalent (GAE) per g protein added) in milk-based blackcurrant samples with polyphenol-to-protein ratios of 5:40 and 10:40.

At the same polyphenol-to-protein ratios, the amount of blackcurrant polyphenols bound the highest to caseins compared to the whey proteins. These results correlate with findings reported in previous sections that extractability of polyphenols using solvent was relatively easier from whey proteins compared to caseins, as indicated by the stronger and larger amount of interactions of the polyphenols with the caseins in the casein-based blackcurrant samples for the loading efficiency studies. In addition, the majority of the bound polyphenols in the milk-based blackcurrant samples were bound to the casein fraction of the milk proteins, as shown in Figure 3. Hence, the preferential binding of blackcurrant polyphenols to milk proteins was casein > milk proteins (combination of caseins and whey proteins) > whey protein. Casein proteins contain both hydrophobic and hydrophilic regions and have a porous structure with cavities and channels that allow them to entrap more polyphenols to form stable complexes or conjugates as compared to whey proteins [30]. These unique structural properties also make caseins appropriate for delivering other bioactive components and drugs, including vitamins, omega-3 fatty acids, and drug molecules, including benzydamine, celecoxib, doxorubicin, ibuprofen, metformin, paclitaxel, and ritonavir [31,32,33,34,35,36,37,38,39,40,41]. The order of protein affinity towards tea polyphenols was caseins > β-lactoglobulin, as reported by Chanphai et al. [42]. In this present study, we analyzed 17 types of individual polyphenols in blackcurrant extracts to determine the competition of polyphenols for binding with milk proteins and the results indicated that caseins preferentially interact with rutin, cyanidin-3-rutinoside, procyanidin B1, delphinidin-3-rutinoside, and gallic acid (descending order), whereas whey proteins preferentially bind caffeic acid, rutin, gallic acid, protocatechuic acid, and catechin (descending order) at the higher concentration of polyphenol-to-protein ratio of 10:40 (Supplementary Figures S1 and S2). When casein and whey protein fractions were both present in the milk-based blackcurrant samples, polyphenols were more likely to bind with the casein fraction rather than the whey protein fraction and showed similar preference to binding rutin and cyanidin-3-rutinoside (descending order) as observed for the casein-based blackcurrant samples, further proving the preferential binding of polyphenols to casein. Moreover, the quantity of bound polyphenols increased with the increase in polyphenol concentration in the samples.

**Figure 4:**
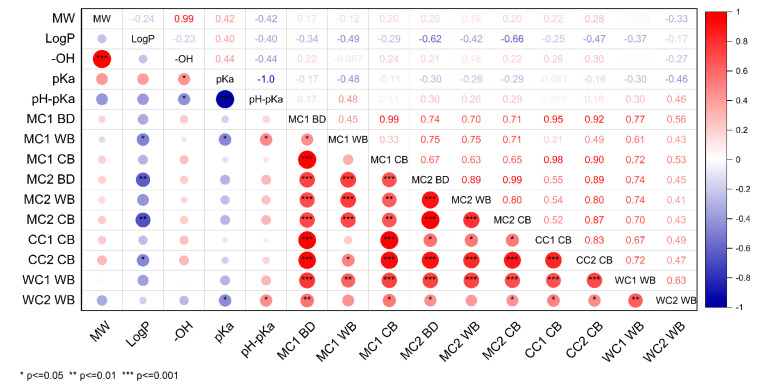
Correlation analysis of the parameters, including molecular weight (MW), the degree of hydrophobicity (logP), hydroxyl group numbers (-OH), pKa, charge (pH-pKa), and the amount of the blackcurrant polyphenols bound to proteins in milk (M)-, whey protein (W)-, casein (C)-based blackcurrant samples, in polyphenol-to-protein ratios of 5:40 (C1) and 10:40 (C2). Specifically, MC1, MC1 WB, and MC1 CB represent the amount of the blackcurrant polyphenols bound to total protein, whey protein, and casein fractions in milk-based blackcurrant samples with a polyphenol-to-protein ratio of 5:40, respectively. MC2, MC2 WB, and MC2 CB represent the amount of the blackcurrant polyphenols bound to total protein, whey protein, and casein fractions in milk-based blackcurrant samples with a polyphenol-to-protein ratio of 10:40, respectively. CC1 and CC2 represent the amount of the polyphenols bound to proteins in casein-based blackcurrant samples in a ratio of 5:40 and 10:40, respectively. WC1 and WC2 represent the amount of the polyphenols bound to proteins in the whey protein-based blackcurrant samples in a ratio of 5:40 and 10:40, respectively.

In order to understand how the parameters of polyphenols investigated in our study, including molecular weight (154.12–934.6 g/mol), logP (−1.7–3.38), hydroxyl group numbers (2–16), pKa (3.27–9), and charge based on pH-pKa (−2.5–3.23), affected the preferential binding and the competition of polyphenols for binding with milk proteins, a correlation analysis was conducted, and the results were illustrated in Figure 4. The value of pH-pKa reflects the charge of the polyphenols, with a positive value signifying a positive charge on the ligand and a negative value indicating a negative charge [43]. Our results demonstrate that the hydrophobicity of polyphenols (logP) is negatively correlated with the amount of polyphenols bound to milk proteins, including casein and a combination of casein and whey proteins at a high polyphenol-to-protein ratio of 10:40 (*p* ≤ 0.01). In other words, the lower the hydrophobicity of the polyphenol, the higher the amount of polyphenols bound to milk proteins. This is likely explained by the four predominant polyphenols in blackcurrant having logP ranging between −1.7 to −0.64), increasing the possibility of hydrogen bonds as the predominant interaction for the current study. On the contrary, our previous study evaluating factors affecting the interaction between various polyphenols from different sources and milk protein indicated that hydrophobic interactions play a major role as opposed to hydrogen bond and electrostatic interactions [11]. This difference is owing to the predominant role of hydrophilic rutin and rutin-based anthocyanins in blackcurrant extracts investigated in this study. No relationship was found between the number of the hydroxyl groups in polyphenols with the bound polyphenols to different fractions of milk proteins (*p* > 0.5), as there is an impact of the distribution of the hydroxyl groups on the different rings (A, B, and C) in the polyphenol structures [11]. However, more studies are needed to understand the specific impact of the position of the hydroxyl group of polyphenols on complex formation with milk proteins. In addition, the pKa of polyphenols was negatively related to the quantity of polyphenols bound to whey proteins in both milk- and whey protein-based blackcurrant samples (*p* ≤ 0.5). Conversely, the value of pH-pKa displayed a positive relationship with the total polyphenols bound to whey proteins at a higher polyphenol concentration (*p* ≤ 0.5), suggesting a corresponding increase in the amount of polyphenols bound to whey proteins as the positive charge of polyphenols increased. This observation underscores the significance of electrostatic interactions in the binding of polyphenols to whey proteins. However, no significant results were found relating to the relationship between change in polyphenols and casein (*p* > 0.5). Moreover, the molecular weight of polyphenols was also not related to the binding efficiency of polyphenols to milk proteins (*p* > 0.5). 

Overall, polyphenols from blackcurrant extracts preferentially bind to the casein fraction in the milk proteins compared to the whey protein. The logP of the polyphenols was inversely related to the amount of polyphenols bound to milk proteins, as the main polyphenols in the blackcurrant possessed a large amount of hydroxyl groups. In addition, the electrostatic interactions played an important role in the polyphenol–whey protein interactions as the charge on polyphenols was positively related to the amount of polyphenols bound to whey proteins. These findings emphasize the selective binding of polyphenols to various milk protein fractions and provide options for the dairy industry to consider during the development of functional dairy products to incorporate the health benefits of specific polyphenols, enabling maximum utilization of polyphenols even in mixed systems. Further research investigating the saturation point of milk proteins for binding with different polyphenols is still needed.

### 3.3. FT-IR Spectra of Polyphenol-Milk Protein System

FT-IR was used to determine whether milk proteins underwent secondary structural changes as a result of their interaction with the polyphenol in the blackcurrant extract, and the results were presented as spectra with different shifts and intensities. The protein amide I, II, A, and B bands were discussed in the absence and presence of polyphenols. The bands of amide I (1700–1600 cm^−1^) and II (1600–1500 cm^−1^) are two main bands of the infrared spectrum of proteins, which are mainly related to the stretching vibration of C=O and in-plane N-H bending coupled with C-N groups, respectively [44]. The amide A band results mainly from the N-H stretching vibration and occurs in the wavenumbers range between 3225 and 3280 cm^−1^, while the amide B band is due to the stretching vibration of C-H and N-H groups and corresponds to the region around 2900 cm^−1^ [45]. Other protein bands with obvious changes after interacting with milk proteins were also evaluated.

**Figure 5:**
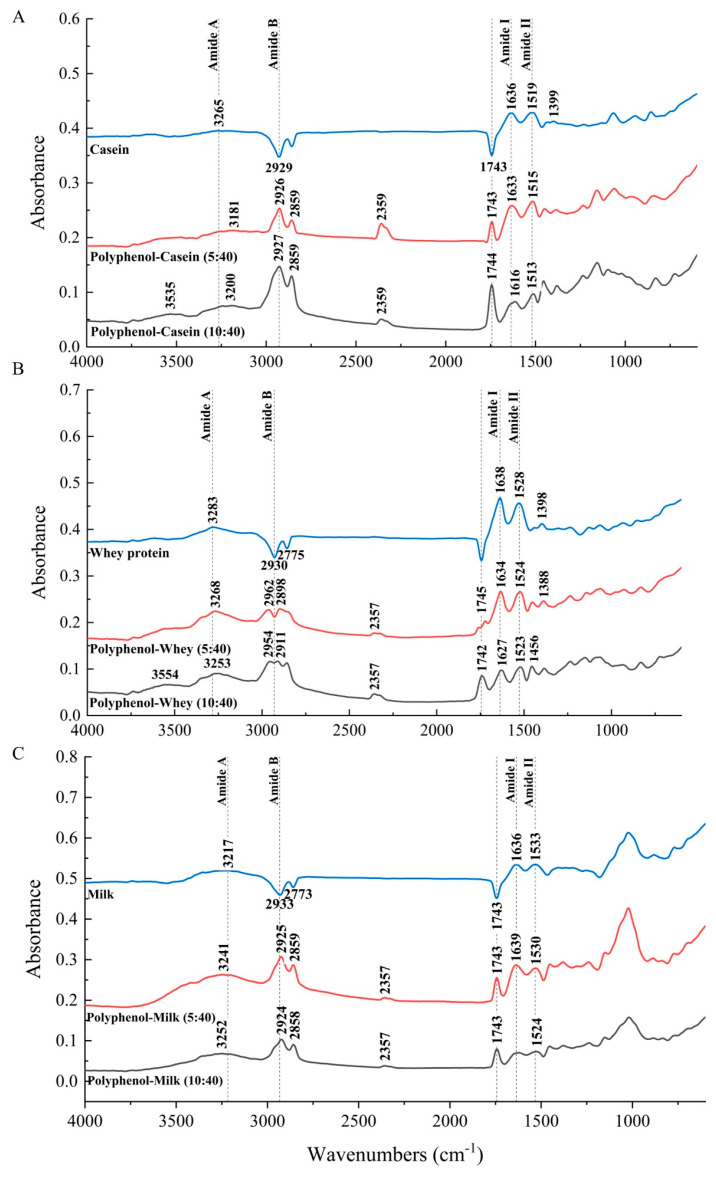
FT-IR spectra of casein (**A**), whey protein (**B**), and milk (**C**) in the absence and presence of polyphenol at room temperature.

**Figure 6:**
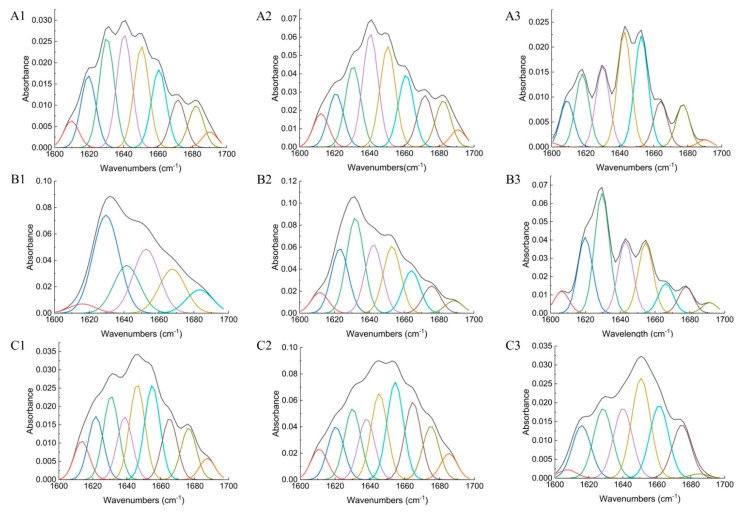
Second derivative analysis and curve-fitted amide I region (1700–1600 cm^−1^) of FT-IR spectroscopy for casein (**A**), whey protein (**B**), and milk (**C**) for protein-alone control sample (1), and protein-based blackcurrant samples with a ratio of blackcurrant polyphenol-to-milk proteins of 5:40 (2) and 10:40 (3).

The infrared spectra of whey proteins in whey protein-based blackcurrant samples in the absence and presence of different concentrations of polyphenol with polyphenol-to-protein ratios of 5:40 and 10:40 at room temperature were shown in Figure 5. When the polyphenol extract concentration was low (5:40), the amide I/II peak migrated from 1636 to 1633 cm^−1^ and 1519 to 1515 cm^−1^. A more obvious migration was found in whey protein amide I and II peaks after increasing polyphenol content (10:40). These findings verified the interaction of polyphenols with whey proteins by hydrogen bonds and electrostatic interactions [42]. This is also consistent with our correlation analysis, where we found that the charge on the polyphenols was positively related to the amount of polyphenols bound to whey proteins, indicating the role of electrostatic interactions. The shift of the amide A peak to lower frequencies on both polyphenol concentrations and the strong peak that appeared in the amide B bond further proved the role of hydrogen bonds in binding polyphenols to milk proteins. A peak around 1743 cm^−1^ also provided evidence that polyphenols interacted with whey protein electrostatically. Moreover, the intensity of whey protein amide I and II was decreased after polyphenols were added to whey protein with higher polyphenol concentration exhibiting lower intensity, suggesting polyphenols were binding to whey proteins hydrophobically. Quantitative analysis of secondary structure change in whey protein after interacting with blackcurrant polyphenols (Table 1 and Figure 6) also identified a significant increase in β-sheet structure (*p* < 0.0001) and a decrease in random coil structures (*p* = 0.001) when the concentration of blackcurrant polyphenols was high, suggesting a more organized secondary structure formed after interacting with blackcurrant polyphenols [46]. The changes were not pronounced when the blackcurrant polyphenols concentration was low (*p* = 0.076). No significant difference was detected regarding α-helix and β-turn structures in the presence of either high or low concentrations of blackcurrant polyphenols. This lack of difference could be explained by the interactions between polyphenols and whey protein reaching their maximum at the 5:40 ratio.

The infrared spectra of casein in casein-based blackcurrant samples before and after the attachment of polyphenols for samples with polyphenol-to-protein ratios of 5:40 and 10:40 at room temperature are displayed in Figure 5. When the concentration of polyphenols was low (5:40), the amide I peak shifted from 1636 cm^−1^ to 1633 cm^−1^, and the amide II peak shifted from 1519 cm^−1^ to 1515 cm^−1^. As the polyphenol concentration increased in the sample (10:40), there were more pronounced shifts in the amide I and amide II bands, shifting from 1636 cm^−1^ to 1616 cm^−1^ and 1519 cm^−1^ to 1513 cm^−1^, respectively. The shift to lower frequencies was due to the formation of hydrogen bonding and electrostatic interactions between –NH_3_^+^ of the caseins and -COO- groups of polyphenols [47,48,49]. Further evidence for electrostatic interactions can be found in the presence of a peak around 1743 cm^−1^ (C=O stretch). The shifting of the amide A band (N-H stretch) following binding to polyphenol at low and high concentrations, from 3265 cm^−1^ to 3181 cm^−1^ and 3200 cm^−1^, respectively, also revealed the role of casein NH groups in the formation of hydrogen bonds and electrostatic interactions with polyphenols [47]. In addition, there was a strong broadening of the peak in the amide B band in the casein polyphenol system with both concentrations, further indicating that hydrogen bonds existed in the interaction between casein and blackcurrant polyphenol extract. The intensity of casein amide I and amide II bands decreased after the attachment of polyphenols, indicating that polyphenols were binding to caseins hydrophobically. Furthermore, quantitative analysis of the secondary structure change in casein in the presence of a low concentration of blackcurrant polyphenols showed a significant decrease in casein β-sheet from 46.43% to 40.98% (*p* = 0.0048), potentially indicating the exposure of hydrophobic related site(s) and enhanced surface hydrophobicity of casein [50,51] (Table 1 and Figure 6). Similar infrared spectral changes were also reported by Hasni, Bourassa, Hamdani, Samson, Carpentier, and Tajmir-Riahi [49], who observed a major reduction in casein β-sheet and α-helical structures in the presence of tea polyphenols. After increasing the concentration of polyphenols, the β-sheet structure continued to decrease, indicating the unfolding of casein structures after interacting with blackcurrant polyphenols (Table 1).

The infrared spectrum of milk protein in milk-based blackcurrant samples before and after interaction with polyphenols at the polyphenol-to-protein ratios of 5:40 and 10:40 is presented in Figure 5. The amide I, II, and A bands migrated after adding polyphenols to milk, indicating the interaction of polyphenols with milk proteins by hydrogen bonds and electrostatic interactions. The presence of electrostatic interactions in the formation of polyphenol–milk protein complexes was further demonstrated by the peak near 1745 cm^−1^. Also, the presence of an amide B peak proved additional evidence that hydrogen bonds were formed when the polyphenols were attached to milk proteins. In terms of the intensity of amide I and II bands, a notable decrease was found after adding polyphenol, indicating polyphenol was binding to milk proteins hydrophobically. In addition, the quantitative analysis of secondary structure change in milk proteins after binding with blackcurrant polyphenols also demonstrated a significant increase in the α-helical structure and a decrease in β-sheet structures, and this change differed with the concentration of blackcurrant polyphenols (Table 1 and Figure 6).

In conclusion, the polyphenols from blackcurrant extracts interacted with whey protein, casein, and milk proteins (including both casein and whey protein fractions) mainly via hydrogen bonding, hydrophobic interactions, and electrostatic interactions, regardless of the concentration of polyphenol added, leading to the structural change in proteins. These observations align with our previous findings that indicated the predominant role of noncovalent interactions in forming polyphenol–milk protein complexes in the aqueous phase [11] and provide groundwork for modifying the functional properties of proteins by altering their structures via encapsulation or interaction with polyphenols.

### 3.4. Bioaccessibility and Antioxidant Activity of the Polyphenols Interacting with Milk Proteins in the Gastric Phase

**Figure 7:**
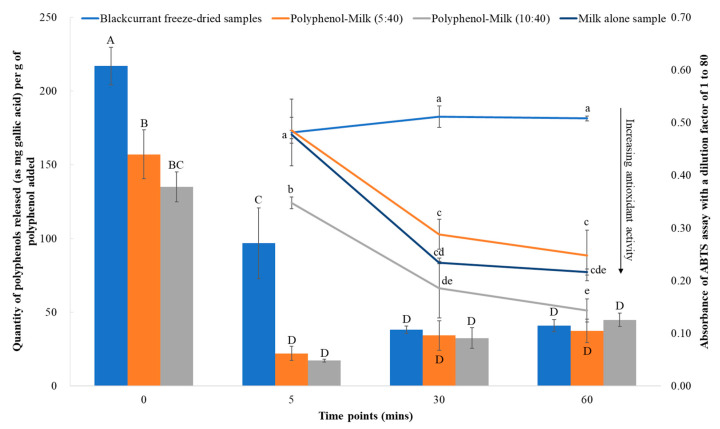
The total phenolic content of *in vitro* gastric phase digests (supernatant) of milk-based blackcurrant samples studied using Folin Ciocalteu polyphenol assay, expressed as the polyphenols released as a fraction of the total polyphenols added initially in the samples based on the Oxi-fend specific sheet (clustered columns in the figure). Antioxidant activity of *in vitro* gastric phase digests of protein-based blackcurrant samples was studied using ABTS assay, expressed as absorbance observed with a dilution factor of 1 in 80 parts, with the absorbance being inversely related to the antioxidant activity. The blackcurrant freeze-dried control samples were more concentrated than the protein-based blackcurrant samples, with a dilution factor of 1 in 16 parts. The *in vitro* gastric phase digests at various points (30 and 60 min of gastric digestion at pH 2 with pepsin enzyme added, after the ‘5 min’ point, which is before enzyme addition after adjustment of pH to 2) were presented. All of these data are the mean of three treatment replicates. Blue, orange, and gray color in the column indicate blackcurrant freeze-dried samples, blackcurrant polyphenol milk-based samples with a ratio of 5:40, and blackcurrant polyphenol milk-based samples with a ratio of 10:40, respectively. Different uppercase and lowercase letters indicate significant differences within the values of columns or lines, respectively (*p* < 0.05).

The bioaccessibility of the polyphenols interacting with milk proteins in milk-based blackcurrant samples in the gastric phase was evaluated by studying the TPC and antioxidant activity of the samples in simulated *in vitro* gastric digestion conditions. The results indicated that the TPC of the blackcurrant-alone samples decreased considerably in the acidic pH conditions of the gastric phase, indicating their rapid degradation (Figure 7). A significant decrease from 217.02 ± 12.75 to 96.82 ± 24.02 mg GAE per g of polyphenol added was observed after adjusting pH from 6.5 to 2 in 5 min (*p* < 0.001). The reduction was more notable after 60 min compared to the initial state (0 min), with the TPC decrease to 40.96 ± 4.11 mg GAE per g of polyphenol added (*p* < 0.001). This is also supported by the lower antioxidant activity at 60 min (gastric phase) of digestion for the blackcurrant-alone samples compared to milk-based blackcurrant samples (*p* < 0.5). In terms of the milk-based blackcurrant samples, the free polyphenols at 0 min time point were significantly lower than the polyphenol-alone samples for both polyphenol-to-protein ratios of 5:40 and 10:40, reflecting the strong interaction of polyphenols with milk proteins, which reduces their release during extraction for TPC analysis. During the later time points of the gastric phase (30 and 60 min), polyphenols were released from milk protein–polyphenol complexes as a result of enzyme proteolysis, and the corresponding antioxidant activity increased significantly compared to the initial state. However, the increase in antioxidant activity for all milk-based samples over the digestion period can be attributed to the reaction of the antioxidant assay with milk protein hydrolysates. The substantial differences in the antioxidant activity observed in the sample with the higher concentration of polyphenols confirmed the impact on the overall antioxidant activity of the milk proteins after their interaction with polyphenols. The antioxidant activity of released polyphenols in the gastric phase exceeded the polyphenol-alone sample at both polyphenol-to-protein ratios of 5:40 and 10:40, suggesting the protective effects of milk proteins to encapsulate or deliver polyphenols. This is consistent with the finding reported by [14], who reported the bioaccessibility of polyphenols from blackcurrant was higher than polyphenol-alone samples after combination with proteins. 

The TPC available at various time points (5, 30, and 60 min) during the gastric digestion process for each sample is illustrated in Figure 7. The polyphenols released from milk-based blackcurrant samples gradually increased, regardless of the polyphenol concentration added in the samples, suggesting the protection and slow release of polyphenols by encapsulating or interacting with milk proteins. No significant difference was observed for polyphenols released in gastric digests for milk-based blackcurrant samples compared to polyphenol-alone samples after 30 min and 60 min digestion. This could be owing to the low release of polyphenols from the milk proteins during the gastric digestion phase, as the extent of proteolysis is very limited compared to the intestinal phase. Pepsin is more effective in breaking down proteins into smaller peptides rather than completely hydrolyzing them into amino acids. Future studies should explore the impact of *in vitro* digestion in the intestinal phase as well to further understand the release of polyphenols during digestion. Other studies have also shown that the bioaccessibility of anthocyanins was enhanced in interaction with caseins and β-lactoglobulin when studied for *in vitro* digestion conditions [52,53]. Therefore, the interaction of polyphenols with milk proteins can protect against the degradation of polyphenols and inhibit the degradation of polyphenols in the gastric phase. 

Overall, the bioaccessibility and antioxidant activity of polyphenols was enhanced as a result of interaction with milk proteins when exposed to the gastric phase digestion process. There was evident interaction of milk protein with Trolox; hence, the common practice of using the Trolox standard curve to express the antioxidant activity of polyphenol–milk protein complexes is not appropriate, and many previous studies will need to be re-evaluated. To further understand the slow and controlled release of polyphenols, intestinal digestion conditions need to be studied.

## 4. Conclusions

The health benefits of polyphenols are well known and accepted. However, their low bioaccessibility and high degradation rates in digestion conditions are broadly ignored in most nutraceutical studies to deliver polyphenols into the diet. Our findings show that the major polyphenols in blackcurrant extract are a variety of anthocyanins, which are preferentially bound with caseins, followed by a mix of casein and whey protein fractions in milk and least to whey proteins. As most of the major polyphenols studied in blackcurrant extracts have low hydrophobicity and a more hydrophilic nature, there is an inverse correlation of hydrophobicity with the binding of the polyphenols to both casein and whey protein. In addition, there was a positive relationship between the charge of polyphenols and their amount bound to whey proteins. Moreover, blackcurrant polyphenols noncovalently interact with milk proteins via hydrogen bonding, hydrophobic, and electrostatic interactions. These interactions with polyphenols induced secondary structural changes in milk proteins, including a notable decrease in β-sheet content in caseins, a significant increase in β-sheet content, and a decrease in random coil content in whey proteins. The interaction of polyphenols with milk proteins also prevents their degradation and controls the release of polyphenols during the gastric digestion phase. Moreover, the bioaccessibility and antioxidant activity of polyphenols were enhanced when the polyphenols interacted with and were delivered by milk proteins at polyphenol-to-protein ratios of 5:40 and 10:40 compared to polyphenol-alone samples. Notably, the interference of Trolox with milk proteins should be emphasized during the antioxidant analysis of polyphenols in the interaction with milk proteins. These observations emphasize the potential for the use of milk proteins to deliver polyphenols in an aqueous phase, paving the way to develop functional dairy foods that incorporate the health benefits of polyphenols. However, more studies are required regarding the role of the nature of polyphenols, including the degree of hydrophobicity and surface charge, in the interaction of various milk proteins. In the future, we should collate a database on the quenching and binding affinities of polyphenols with milk proteins, using different mixed systems with varied ratios of polyphenols and proteins. To better understand the interaction forces as well as the degrees, other methods such as UV-vis spectroscopy, fluorescence spectroscopy, isothermal titration calorimetry, and molecular docking simulation should be considered. Further *in vitro* intestinal phase studies are also needed to confirm the controlled release of polyphenols from milk proteins, in addition to in vivo, animal, and clinical studies.

## Figures and Tables

**Table 1:** FT-IR analysis of the secondary structure of casein-, whey protein-, and milk-based blackcurrant samples with polyphenol-to-protein ratios of 5:40 and 10:40 by second derivative method ^1^.

	β-Sheet/%	Random Roil/%	α-Helix/%	β-Turn/%
Casein	46.43 ± 2.85 ^b^	8.08 ± 0.94 ^a^	29.21 ± 3.65 ^bc^	16.28 ± 1.74 ^b^
Blackcurrant–Casein (5:40)	40.98 ± 1.23 ^c^	5.58 ± 1.28 ^b^	25.29 ± 1.91 ^c^	28.14 ± 3.41 ^a^
Blackcurrant–Casein (10:40)	39.98 ± 1.7 ^c^	6.05 ± 1.59 ^b^	24.64 ± 1.91 ^c^	29.33 ± 3.16 ^a^
Whey	46.71 ± 1.36 ^b^	8.05 ± 0.65 ^a^	27.23 ± 0.56 ^c^	18.01 ± 0.41 ^b^
Blackcurrant–Whey (5:40)	49.37 ± 1.5 ^b^	7.44 ± 0.33 ^a^	27.3 ± 2.15 ^c^	15.88 ± 3.36 ^b^
Blackcurrant–Whey (10:40)	53.22 ± 2.54 ^a^	5.19 ± 0.89 ^b^	26.91 ± 2.82 ^c^	14.68 ± 4.04 ^b^
Milk	48.54 ± 1.71 ^b^	5.95 ± 1.02 ^b^	27.38 ± 0.46 ^bc^	18.14 ± 0.41 ^b^
Blackcurrant–Milk (5:40)	39.77 ± 1.14 ^c^	7.51 ± 0.15 ^a^	34.72 ± 3.55 ^a^	17.99 ± 4.41 ^b^
Blackcurrant–Milk (10:40)	40.99 ± 2 ^c^	6.2 ± 0.82 ^a^	35.15 ± 1.53 ^a^	17.65 ± 2.4 ^b^

^1^ Different superscript indicates significant differences within the values of each column (*p* < 0.05).

## Data Availability

Data is contained within the article or supplementary material.

## Supplementary Materials

The following supporting information can be downloaded at: https://www.mdpi.com/article/10.3390/foods13040515/s1.
