# Supplementary material for: Preferential Binding of Polyphenols in Blackcurrant Extracts with Milk Proteins and the Effects on the Bioaccessibility and Antioxidant Activity of Polyphenols
Source: Foods. 2024 Feb 7;13(4):515. doi: 10.3390/foods13040515 (PMC10887666; doi:10.3390/foods13040515)
Supplement: Supplementary file 1 [file foods-13-00515-s001.zip › foods-2857469-supplementary.pdf]

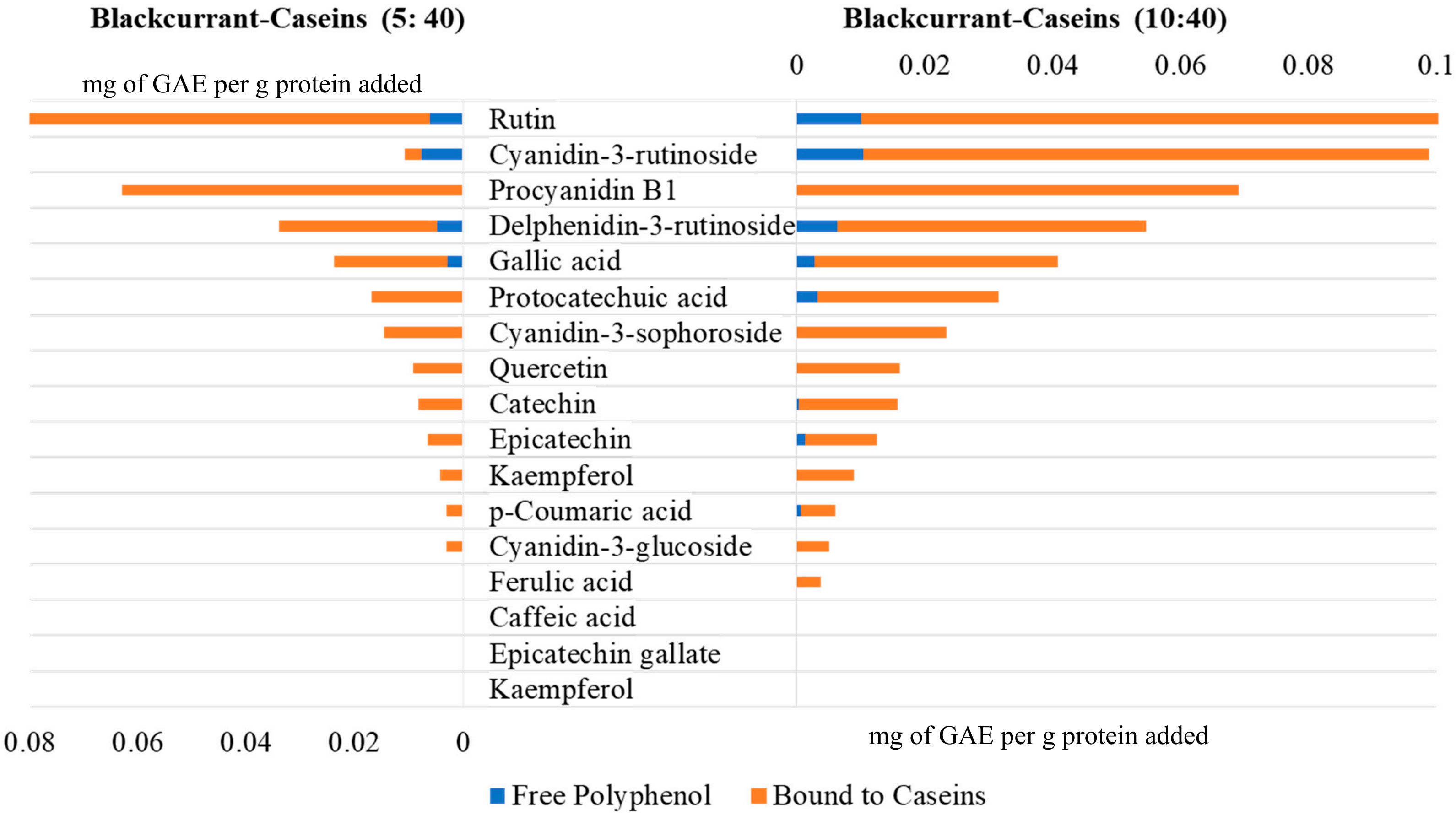

Supplementary Figure S1. Free and bound polyphenol content (expressed as mg of gallic acid equivalent (GAE) per g protein added) in casein-based blackcurrant samples with a ratio of polyphenol to caseins of 5:40 and 10:40.

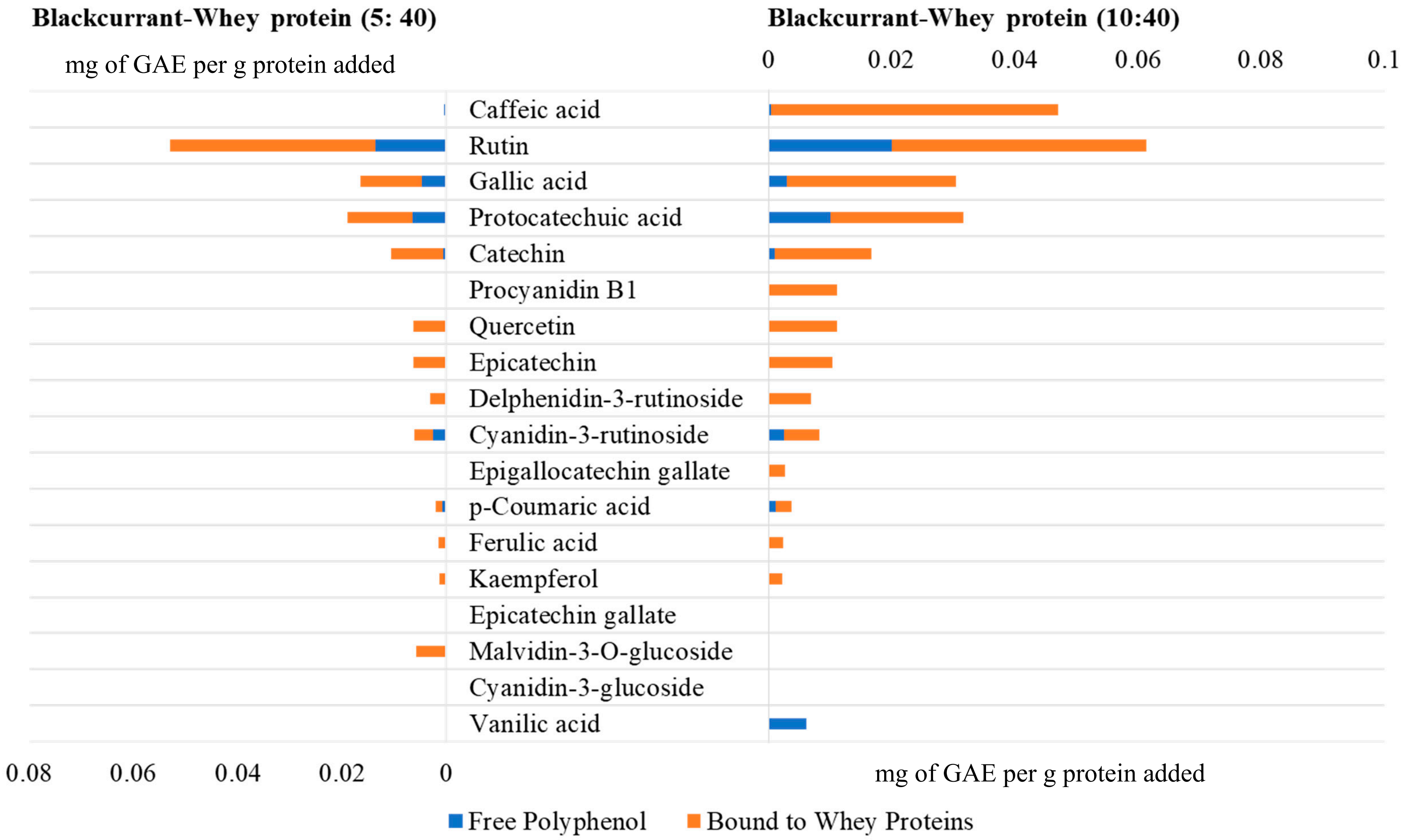

Supplementary Figure S2. Free and bound polyphenol content (expressed as mg of gallic acid equivalent (GAE) per g protein added) in whey protein-based blackcurrant samples with a ratio of polyphenol to whey protein of 5:40 and 10:40.
